# Prediction of protein-protein interactions from amino acid sequences with ensemble extreme learning machines and principal component analysis

**DOI:** 10.1186/1471-2105-14-S8-S10

**Published:** 2013-05-09

**Authors:** Zhu-Hong You, Ying-Ke Lei, Lin Zhu, Junfeng Xia, Bing Wang

**Affiliations:** 1 College of Computer Science and Software Engineering, Shenzhen University, Shenzhen, Guangdong 518060, China; 2 Department of Information, Electronic Engineering Institute, Hefei, Anhui 230601, China; 3 Department of Automation, Un iversity of Science and Technology of China, Hefei, Anhui 230601, China; 4 Institute of Health Sciences, Anhui University, Hefei, Anhui 230601, China; 5 The Advanced Research Institute of Intelligent Sensing Network, Tongji University, shanghai, 201804, China

## Abstract

**Background:**

Protein-protein interactions (PPIs) play crucial roles in the execution of various cellular processes and form the basis of biological mechanisms. Although large amount of PPIs data for different species has been generated by high-throughput experimental techniques, current PPI pairs obtained with experimental methods cover only a fraction of the complete PPI networks, and further, the experimental methods for identifying PPIs are both time-consuming and expensive. Hence, it is urgent and challenging to develop automated computational methods to efficiently and accurately predict PPIs.

**Results:**

We present here a novel hierarchical PCA-EELM (principal component analysis-ensemble extreme learning machine) model to predict protein-protein interactions only using the information of protein sequences. In the proposed method, 11188 protein pairs retrieved from the DIP database were encoded into feature vectors by using four kinds of protein sequences information. Focusing on dimension reduction, an effective feature extraction method PCA was then employed to construct the most discriminative new feature set. Finally, multiple extreme learning machines were trained and then aggregated into a consensus classifier by majority voting. The ensembling of extreme learning machine removes the dependence of results on initial random weights and improves the prediction performance.

**Conclusions:**

When performed on the PPI data of *Saccharomyces cerevisiae*, the proposed method achieved 87.00% prediction accuracy with 86.15% sensitivity at the precision of 87.59%. Extensive experiments are performed to compare our method with state-of-the-art techniques Support Vector Machine (SVM). Experimental results demonstrate that proposed PCA-EELM outperforms the SVM method by 5-fold cross-validation. Besides, PCA-EELM performs faster than PCA-SVM based method. Consequently, the proposed approach can be considered as a new promising and powerful tools for predicting PPI with excellent performance and less time.

## Background

Proteins are crucial for almost all of functions in the cell, including metabolic cycles, DNA transcription and replication, and signalling cascades. Usually, proteins rarely perform their functions alone; instead they cooperate with other proteins by forming a huge network of protein-protein interactions (PPIs) [1]. PPIs are responsible for the majority of cellular functions. In the past decades, many innovative techniques for detecting PPIs have been developed [1-3]. Due to the progress in large-scale experimental technologies such as yeast two-hybrid (Y2H) screens [2,4], tandem affinity purification (TAP) [1], mass spectrometric protein complex identification (MS-PCI) [3] and other high-throughput biological techniques for PPIs detection, a large amount of PPIs data for different species has been accumulated [1-5]. However, the experimental methods are costly and time consuming, therefore current PPI pairs obtained from experiments only covers a small fraction of the complete PPI networks [6]. In addition, large-scale experimental methods usually suffer from high rates of both false positive and false negative predictions [6-8]. Hence, it is of great practical significance to develop the reliable computational methods to facilitate the identification of PPIs [9-11].

A number of computational methods have been proposed for the prediction of PPIs based on different data types, including phylogenetic profiles, gene neighborhood, gene fusion, literature mining knowledge, and sequence conservation between interacting proteins [6-9,12-15]. There are also methods that combine interaction information from several different data sources [16]. However, these methods cannot be implemented if such pre-knowledge about the proteins is not available. Recently, a couple of methods which derive information directly from amino acid sequence are of particular interest [7-9,11]. Many researchers have engaged in the development of sequences-based method for discovering new PPIs, and the experiment results showed that the information of amino acid sequences alone is sufficient to predict PPIs[7,9,11]. Among them, one of the excellent works is a SVM-based method developed by Shen et al [11]. In the study, the 20 amino acids were clustered into seven classes according to their dipoles and volumes of the side chains, and then the conjoint triad method abstracts the features of protein pairs based on the classification of amino acids. When applied to predict human PPIs, this method yields a high prediction accuracy of 83.9%. Because the conjoint triad method cannot takes neighboring effect into account and the interactions usually occur in the discontinuous amino acids segments in the sequence, on the other work Guo et al. developed a method based on SVM and auto covariance to extract the interactions information in the discontinuous amino acids segments in the sequence [9]. Their method yielded a prediction accuracy of 86.55%, when applied to predicting *saccharomyces cerevisiae *PPIs. In our previous works, we also obtained good prediction performance by using autocorrelation descriptors and correlation coefficient, respectively [8,17].

The general trend in current study for predicting PPIs has focused on high accuracy but has not considered the time taken to train the classification models, which should be an important factor of developing a sequence-based method for predicting PPIs because the total number of possible PPIs is very large. Therefore some computational models with high classification accuracy may not be satisfactory when considering the trade-off between the classification accuracy and the time for training the models. Recently, Huang et al. proposed a new learning algorithm called extreme learning machine (ELM), which randomly assigns all the hidden node parameters of generalized single-hidden layer feed-forward networks (SLFNs) and analytically determines the output weights of SLFNs[18-21]. Previous works shown that ELM provides efficient unified solutions to generalized feed-forward networks including kernel learning. Consequently, ELM offers significant advantages such as fast learning speed, ease of implementation, and least human intervention. ELM has good potential as a viable alternative technique for large-scale computing and artificial intelligence. On the other hand, single ELM model is sometime difficult to achieve a satisfactory performance for the complex processes with strong nonlinearity, time variant and highly uncertainty. Ensemble ELM methods have received special attentions because it can improve the accuracy of predictor and achieve better stability through training a set of models and then combining them for final predictions [22-24]. For example, Lan et al. proposed an ensemble of online sequential ELM with more stable and accurate results [25]. Zhao et al. proposed an ensemble ELM soft sensing model for effluent quality prediction based on kernel principal component analysis (KPCA), whose reliability and accuracy outperforms other models [24]. In this study, an ensemble ELM model was built to predict the protein interactions.

Previous works have pointed out that using feature selection or feature extraction before conducting the classification tasks can improve the classification accuracy[26]. Here, we attempt to examine the effectiveness of the dimensionality reduction technique before constructing the ELM classifier for the PPI prediction. Principal component analysis (PCA) is utilized to do the feature extraction which projects the original feature space into a new space, on which the ELM is used to perform the prediction task. The effectiveness of the proposed PCA-ELM is examined in terms of classification accuracy on the PPI dataset. Promisingly, as can be seen that the developed PCA-ELM PPI prediction system has achieved high accuracy and runs very fast as well.

In this study, we report a new sequence-based method for the prediction of protein-protein interactions from amino acid sequences with ensemble ELM and PCA aiming at improving the efficiency and effectiveness of the classification accuracy. Firstly, four kinds of useful sequence-based features such as Auto Covariance (AC), Conjoint triad (CT), Local descriptor (LD) and Moran autocorrelation (MAC) are extracted from each protein sequence to mine the interaction information in the sequence. Secondly, in order to reduce the computational complexity and enhance the overall accuracy of the predictor, an effective feature reduction method PCA is employed to extract the most discriminative new feature subset. Finally, ELM is chosen as the weak learning machine and the ensemble ELM classifier is constructed using the vectors of resulting feature subset as input. To evaluate the performance, the proposed method was applied to *Saccharomyces cerevisiae *PPI data. The experiment results show that our method achieved 87% prediction accuracy with 86.15% sensitivity at the precision of 87.59%. The prediction model was also assessed using the independent dataset of the *Escherichia coli *PPIs and yielded 87.5% prediction accuracy, which further demonstrates the effectiveness of our method.

## Results

In this section, we first discuss the biological datasets and evaluation strategies used in performance comparisons. Next we present results for comparing the PCA-EELM method to state-of-the-art classifier for predicting protein interaction pairs in yeast.

### Generation of the data set

We evaluated the proposed method with the dataset of physical protein interactions from yeast used in the study of Guo et al. [9]. The PPI dataset was collected from *Saccharomyces cerevisiae *core subset of database of interacting proteins (DIP), version DIP 20070219. After the redundant protein pairs which contain a protein with fewer than 50 residues or have ≥40% sequence identity were remove, the remaining 5594 protein pairs comprise the final positive dataset. The 5594 non-interacting protein pairs were generated from pairs of proteins whose sub-cellular localizations are different. The whole dataset consists of 11188 protein pairs, where half are from the positive dataset and half are from the negative dataset.

### Evaluation measures

To measure the performance of the proposed method, we adopted 5-fold cross validation and four parameters, the overall prediction accuracy (Accu.), sensitivity (Sens.), precision (Prec.) and Matthews correlation coefficient (MCC). They are defined as follows:

(1) $$ACC=\frac{TP+TN}{TP+FP+TN+FN}$$

(2) $$SN=\frac{TP}{TP+FN}$$

(3) $$PE=\frac{TP}{TP+FP}$$

(4) $$MCC=\frac{TP\times TN-FP\times FN}{\sqrt{\left(TP+FN\right)\times \left(TN+FP\right)\times \left(TP+FP\right)\times \left(TN+FN\right)}}$$

where true positive (TP) is the number of true PPIs that are predicted correctly; false negative (FN) is the number of true PPIs that are predicted to be non-interacting pairs; false positive (FP) is the number of true non-interacting pairs that are predicted to be PPIs, and true negative (TN) is the number of true non-interacting pairs that are predicted correctly. MCC denotes Mathews correlation coefficient.

### Experimental setting

The proposed PCA-EELM protein interaction prediction method was implemented using MATLAB platform. For ELM, the implementation by Zhu and Huang available from http://www.ntu.edu.sg/home/egbhuang was used. Regarding SVM, LIBSVM implementation available from http://www.csie.ntu.edu.tw/~cjlin/libsvm was utilized, which was originally developed by Chang and Lin. All the simulations were carried out on a computer with 3.1 GHz 2-core CPU, 6 GB memory and Windows operating system.

All ELM in the ensemble classifier had the same number of hidden layer neurons but different random hidden layer weights and output layer weights. Ensemble ELM models were built via the stratified 5-fold cross-validation procedure through increasing gradually the number of hidden neurons from 20 to 300 in interval of 10. The best number of neurons was adapted to create the training model. The sigmoid activation function was used to compute the hidden layer output matrix. The final model was an ensemble of 15 extreme learning machines, and the outputs of ensemble ELM model were determined by combining the outputs of the each individual ELM by majority voting. For SVM, the Radial Basis Function was chosen as the kernel function and the optimized parameters (C,γ) were obtained with a grid search approach.

### Prediction performance of PCA-EELM model

We evaluated the performance of the proposed PCA-EELM model using the DIP PPIs data as investigated in Guo *et al*. [9]. In order to evaluate the prediction ability of our ELM classifiers, we also implemented a Support Vector Machine (SVM) learning algorithm which is thought of as the state-of-the-art classifier. We have compared our ensemble ELM based recognition scheme against methods utilizing SVM with C = 8, g = 0.5, λ = 30. For the ensemble ELM and SVM classifiers, all of the input values were normalized in the range of [-1,1]. To reduce the bias of training and testing data, a 5-fold cross-validation technique is adopted. More specifically, the dataset is divided into 5 subsets, and the holdout method is reiterated 5 times. Each time four of the five subsets are put together as the training dataset, and the other one subset is utilized for testing the model. Thus five models were generated for the five sets of data. Table 1 demonstrates the average prediction performance of the PCA-EELM and the PCA-SVM modelacross five runs.

**Table 1:** The prediction performance comparison of PCA-EELM with PCA-SVM

Classification Model	Test set	Sens. (%)	Prec. (%)	Accu. (%)	MCC (%)	Testing Time (Seconds)
PCA-EELM	1	86.60	87.32	87.03	77.42	45.1482
	2	85.97	87.34	86.77	77.04	45.4615
	3	85.95	87.68	86.95	77.30	46.7825
	4	86.60	88.10	87.47	78.07	43.2015
	5	85.64	87.52	86.73	76.97	44.1237
	Average	**86.15+0.43**	**87.59+0.32**	**87.00+0.29**	**77.36+0.44**	**44.9435+1.36**

PCA-SVM	1	81.76	82.86	82.16	70.69	52.6035
	2	85.77	81.65	83.37	72.25	51.7143
	3	87.52	80.67	83.10	71.78	51.7143
	4	85.77	82.44	83.77	72.79	51.8547
	5	85.48	79.09	81.74	70.09	51.4335
	
	Average	**85.26+2.12**	**81.34+1.51**	**82.82+0.85**	**71.52+1.1**	.**51.8641+0.44**

It can be observed from Table 1 that SVM shows good prediction accuracy in the range of 81.74%-83.77%. For ensmble ELM, high prediction accuracy in the range of 86.73%-87.47% is obtained. To better investigate the prediction ability of our model, we also calculated the values of Sensitivity, Precision, and MCC. From Table 1, we can see that our model gives good prediction performance with an average Sens. value of 86.15%, Prec. value of 87.59% and MCC value of 77.36%. Further, it can also be seen in the Table 1 that the standard deviation of sensitivity, precision, accuracy and MCC are as low as 0.43, 0.32, 0.29 and 0.44 respectively. The results illustrates that the PCA-EELM is an accurate and efficient method for the prediction of PPIs. To sum up, we can readily conclude that the PCA-EELM approach generally outperforms the excellent PCA-SVM model with higher discrimination power for predicting PPIs based the information of protein sequences.

In addition, it is evident from the results presented in Table 1 that the average learning time of the PCA-EELM classifier is 44.94 seconds while the learning time of the SVM model is 51.86 seconds. The proposed ensemble ELM classifier even run faster than the SVM model. Through these analyses, it is obvious that PCA-EELM model is an efficient classification method in comparison with PCA-SVM method. Therefore, we can see clearly that PCA-EELM model is a much more appropriate method for predicting new protein interactions compared with the other methods. Consequently, it makes us be more convinced that the proposed PCA-EELM based method can be very helpful in assisting the biologist to assist in the design and validation of experimental studies and for the prediction of interaction partners. Thus, in the case of real-time implementation of PPIs prediction system, E-ELM classifiers are more appropriate than SVM model. All the analysis shows that our model is an accurate and fast method for the prediction of PPIs.

### Comparing the prediction performance with other methods

In order to highlight the advantage of our model, it was also tested by *Helicobacter pylori *dataset. The *H. pylori *dataset is composed of 2,916 protein pairs (1,458 interacting pair and 1,458 non-interacting pairs) as described by Martin *et al *[27]. This dataset gives a comparison of proposed method with other previous works including phylogenetic bootstrap[28], signature products[27], HKNN[29], ensemble of HKNN[30] and boosting[17]. The results of 10 fold cross-validation over six different methods are shown in Table 2. The average prediction performance, i.e. sensitivity, precision, accuracy and MCC achieved by PCA-EELM predictor, are 88.95%, 86.15%, 87.50% and 78.13%, respectively. It shows that the prediction results for PCA-EELM predictor and the ensemble of HKNN, outperforms other state-of-the-art methods, which highlight that a multiple classifier system is more accurate and robust than a single classifier. We also observed that the proposed method clearly achieves better results compared to other multiple classifier systems (i.e. ensemble of HKNN and Boosting). All these results show that the proposed PCA-EELM classifier not only achieves accurate performance, but also substantially improves precision in the prediction of PPIs.

**Table 2:** Performance comparison of different methods on the H.pylori dataset. Here, N/A means not available.

Methods	SN (%)	PE (%)	ACC (%)	MCC (%)
Phylogenetic bootstrap	69.8	80.2	75.8	N/A
HKNN	86	84	84	N/A
Signature products	79.9	85.7	83.4	N/A
Ensemble of HKNN	86.7	85	86.6	N/A
Boosting	80.37	81.69	79.52	70.64
Proposed method	**88.95**	**86.15**	**87.50**	**78.13**

## Conclusions

In this paper, we have developed an efficient and fast technique for predicting protein interactions from protein amino acids sequences by combining ensemble ELM with PCA. The main aim of the proposed method is to employ the unique features of ELM classifier including better generalization performance, fast learning speed, simpler and without tedious and time-consuming parameter tuning to predict new protein interactions. In order to remove the noise and irrelevant features which affect the protein prediction performance, the PCA was utilized for feature reduction before conducting the ensemble ELM classifier. Experimental results demonstrated that the proposed method performed significantly well in distinguishing interacting and non-interacting protein pairs. It was observed that PCA-EELM achieved the highest classification accuracy of 89% and mean classification accuracy of 88% using 5-fold cross-validation. Meanwhile, comparative study was conducted on the methods of PCA-SVM and PCA-EELM. The experimental results showed that our method significantly outperformed PCA-SVM in terms of classification accuracy with shorter run time.

## Methods

**Figure 1:**
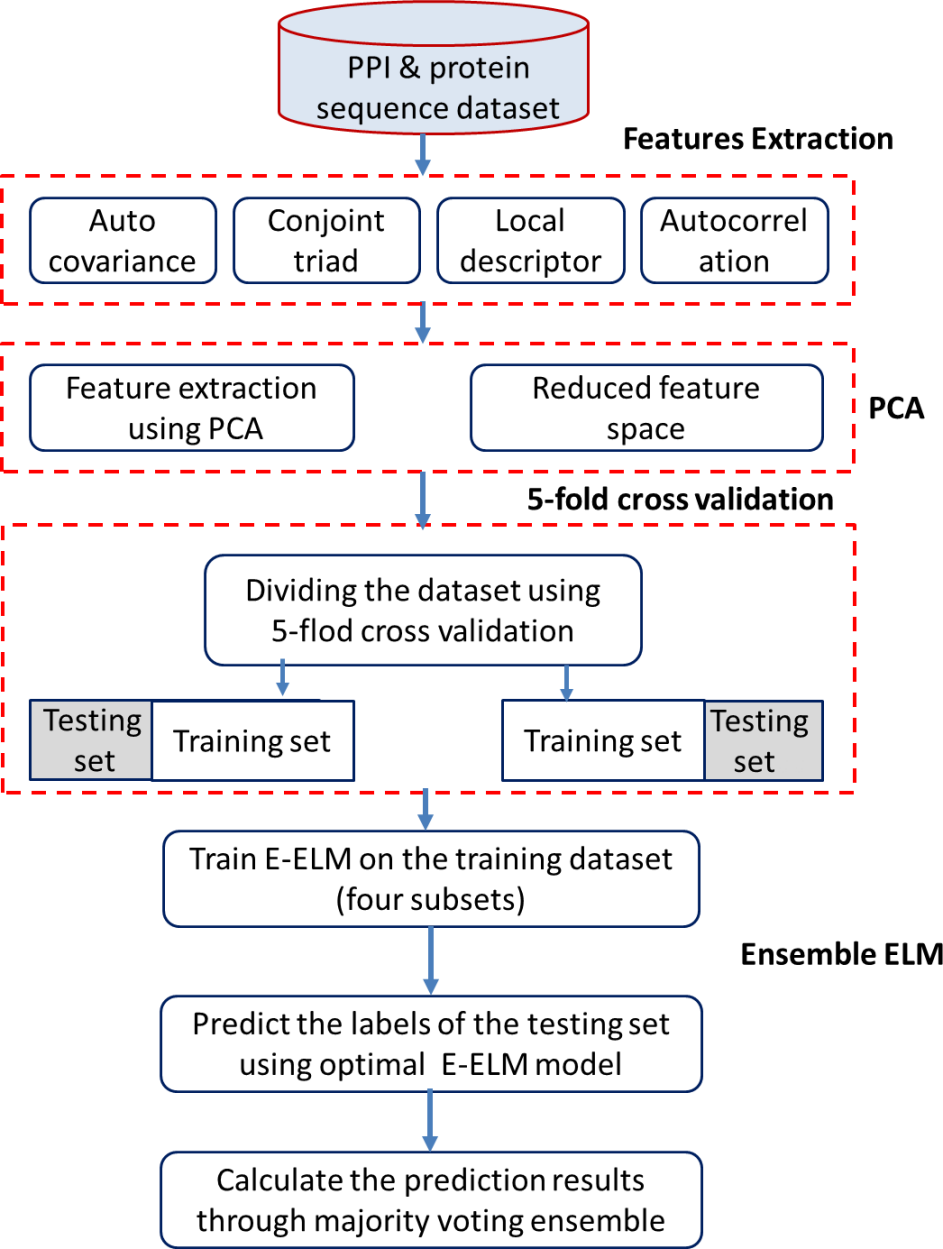
**The architecture of the proposed PCA-EELM protein interaction prediction method**.

In this section, we describe the proposed PCA-EELM approach for predicting protein interactions from protein sequences. The architecture is shown in Figure 1. Our method to predict the PPIs depends on three steps: (1) Represent protein pairs as a vector by using the proposed four kinds of protein sequence descriptors; (2) Principal component analysis is utilized to do the feature reduction; (3) Ensemble ELM is used to perform the protein interaction prediction tasks. In the second stage, dimension reduction is obtained using PCA to project the original feature space into a new space. In the third stage, new feature sets are fed into the ensemble ELM classifier for training an optimal model, meanwhile the number of hidden neurons is chosen which can obtain the most accurate results. Finally, the predict model conducts the protein interaction prediction tasks using the most discriminative new feature set and the optimal parameters.

### Protein sequence representation

To use machine learning methods to predict PPIs from protein sequences, one of the most important computational challenges is to extract feature vectors from protein sequences in which the important information content of proteins is fully encoded. In this study, four kinds of feature representation methods including Auto Covariance (AC), Conjoint triad (CT), Local descriptor (LD) and Moran autocorrelation are employed to transform the protein sequences into feature vectors.

### Auto covariance (AC) scores

Given a protein sequence, auto covariance (AC) accounts for the interactions between amino acids with a certain number of amino acids apart in the sequence, so this method takes neighbouring effect into account and makes it possible to discover patterns that run through entire sequences[9]. Here, six sequence-based physicochemical properties of amino acid were chosen to reflect the amino acids characteristics. These physicochemical properties include hydrophobicity (H), volumes of side chains of amino acids (VSC), polarity (P1), polarizability (P2), solvent-accessible surface area (SASA) and net charge index of side chains (NCISC) of amino acids respectively, which are employed as basis for PPI prediction. Table 3 showed the values of the six physicochemical properties for each amino acid.

**Table 3:** The original values of the six physicochemical properties for each amino acid

Amino acid	H	VSC	P1	P2	SASA	NCISC
A	0.62	27.5	8.1	0.046	1.181	0.007187
C	0.29	44.6	5.5	0.128	1.461	-0.03661
D	-0.9	40	13	0.105	1.587	-0.02382
E	-0.74	62	12.3	0.151	1.862	0.006802
F	1.19	115.5	5.2	0.29	2.228	0.037552
G	0.48	0	9	0	0.881	0.179052
H	-0.4	79	10.4	0.23	2.025	-0.01069
I	1.38	93.5	5.2	0.186	1.81	0.021631
K	-1.5	100	11.3	0.219	2.258	0.017708
L	1.06	93.5	4.9	0.186	1.931	0.051672
M	0.64	94.1	5.7	0.221	2.034	0.002683
N	-0.78	58.7	11.6	0.134	1.655	0.005392
P	0.12	41.9	8	0.131	1.468	0.239531
Q	-0.85	80.7	10.5	0.18	1.932	0.049211
R	-2.53	105	10.5	0.291	2.56	0.043587
S	-0.18	29.3	9.2	0.062	1.298	0.004627
T	-0.05	51.3	8.6	0.108	1.525	0.003352
V	1.08	71.5	5.9	0.14	1.645	0.057004
W	0.81	145.5	5.4	0.409	2.663	0.037977
Y	0.26	117.3	6.2	0.298	2.368	0.023599

H, hydrophobicity; VSC, volume of side chains; P1, polarity; P2, polarizability;

SASA, solvent accessible surface area; NCISC, net charge index of side chains

By this means, the amino acid residues were first translated into numerical values representing physicochemical properties. Then they were normalized to zero mean and unit standard deviation (SD) according to Equation (5):

(5) $$\begin{array}{cc} P_{ij}^{{}^{\prime }}=\frac{P_{ij}-P_{j}}{S_{j}} & \left(i=1,2,...,6;j=1,2,...,20.\right) \end{array}$$

where $P_{ij}$ is the jth descriptor value for ith amino acid, $P_{j}$ is the mean of jth descriptor over the 20 amino acids and $S_{j}$ is the corresponding standard deviation. Then each protein sequence was translated into six vectors with each amino acid represented by the normalized values.

Then auto covariance was used to transform these numerical sequences into uniform matrices. To represent a protein sample P with length L, the AC variables are calculated according to Equation (6):

(6) $$AC\left(\text{l}\;ag,j\right)=\overset{L-lag}{\underset{i=1}{\sum }}\left(P_{i,j}-\frac{1}{L}\overset{L}{\underset{i=1}{\sum }}P_{i,j}\right)\times \left(P_{\left(i+lag\right),j}-\frac{1}{L}\overset{L}{\underset{i=1}{\sum }}P_{i,j}\right)/L-lag)$$

where lag is the distance between residues, j is the jth physicochemical property of nature amino acids mentioned above, i is the position in the sequence P.

In this way, the number of AC variables, D can be calculated as D=lg×q, where q is the number of descriptors and lg is the maximum lag(lag=1,2,...,lg). After each protein sequence was represented as a vector of AC variables, a protein pair was characterized by concatenating the vectors of two proteins in this protein pair.

### Conjoint triad (CT) scores

Conjoint triad (CT) considers the properties of one amino acid and its vicinal amino acids and regards any three continuous amino acids as a unit [11]. Thus, the triad can be differentiated according to the classes of amino acid. The PPI information of protein sequence can be projected into a homogeneous vector space by counting the frequency of each triad type. It should be noted that before using such feature representation method, the 20 amino acids has been clustered into seven classes according to the dipoles and volumes of the side chains. The classification of amino acids is listed in Table 4. And thus the dimensions of a protein sequence were dramatically reduced to 7×7×7 =343. Finally, the descriptors of two proteins were concatenated and a total 686-dimensional vector has been built to represent each protein pair.

**Table 4:** Division of amino acids based on the dipoles and volumes of the side chains

**No**.	Group
1	A, G, V
2	C
3	D, E
4	F, I, L, P
5	H, N, Q, W
6	K, R
7	M, S, T, Y

### Local descriptor (LD) scores

Local descriptor (LD) is an alignment-free approach and its effectiveness depends largely on the underlying amino acid groups [31]. To reduce the complexity inherent in the representation of the 20 standard amino acids, we firstly clustered it into seven functional groups based on the dipoles and volumes of the side chains (see Table 4 for details). Then three local descriptors, Composition (C), Transition (T) and Distribution (D) which is based on the variation of occurrence of functional groups of amino acids within the primary sequence of the protein are calculated. C stands for the composition of each amino acid group along a local region. T represents the percentage frequency with which amino acid in one group is followed by amino acid in another group. D characterizes the distribution pattern along the entire region by measuring the location of the first, 25, 50, 75 and 100% of residues of a given group.

**Figure 2:**
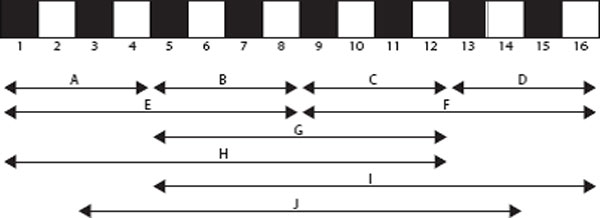
**The 10 regions (A-J) used by the Local Descriptor technique for a theoretical protein sequence**. The regions A-D and E-F are obtained by dividing the entire sequence into four equal regions and into two equal regions respectively. Region G represents the central 50% of the sequence. Regions H, I and J are the first, final and central 75% of the sequence.

In total there would be 63 features (7 composition, 21 transition, 35 distribution) if they were computed from the whole amino acid sequence. However, in order to better capture continuous and discontinuous PPI information from the sequence, we split each protein into 10 local regions(A-J) of varying length and composition as follows: Regions A, B, C and D are obtained by dividing the entire protein sequence into four equal-length regions. Regions E and F are obtained by dividing the protein sequence in two equal-length regions. Region G represents the middle with 50% of the sequence. Region H represents the first 75% of the sequence, Region I the final 75% of the sequence and Region J the middle with 75% of the sequence. These regions are illustrated in Figure 2. For each region the 63 local descriptors are extracted, resulting in a 630 feature vector. Then the PPI pair is characterized by concatenating the two vector spaces of two individual proteins. Thus, a 1260-dimentional vector has been constructed to represent each protein pair and used as a feature vector for input into ELM classifier.

### Autocorrelation scores

Autocorrelation features describe the level of correlation between two protein sequences in terms of their specific physicochemical property, which are defined based on the distribution of amino acid properties along the sequence [8]. There are six amino acid properties used for deriving autocorrelation descriptors as the AC method. Here we use the commonly-used Moran autocorrelation (MAC) to infer PPIs, which can be calculated as:

(7) $$M_{AC}\left(d\right)=\frac{1}{N-d}\overset{N-d}{\underset{j=1}{\sum }}(P_{j}-\bar{P}\times \left(P_{j+d}-\bar{P}\right)/\frac{1}{N}\overset{N}{\underset{j=1}{\sum }}{\left(P_{j}-\bar{P}\right)}^{2})$$

where N is the length of the sequence, d=1,2,...,30 is the distance between on residue and its neighbours, $P_{j}$ and $P_{j+d}$ are the properties of the amino acid at positions j and j+d respectively. $\bar{P}=\overset{N}{\underset{j=1}{\sum }}P_{j}/N)$ is the average value of P.

Therefore, Moran autocorrelation descriptor consists of a total of 30*6 = 180 descriptor values, i.e., a 180-dimensional vector has been built to represent the protein sequence. A representation of an interaction pair is formed by concatenating Moran autocorrelation descriptors of two protein sequences in this protein pairs.

### The feature space

For each protein pair in the dataset, its feature space is composed of the features of Auto Covariance (AC), Conjoint triad (CT), Local descriptor (LD) and Moran autocorrelation (MAC). Totally, there are 2666 features to be encoded in each sample, including 360 MAC features, 1260 LD features, 686 CT features and 360 AC features.

### Principal component analysis (PCA)

PCA is a technique used to reduce multidimensional data sets to lower dimensions for analysis. It is a widely used data analysis technique that allows reducing the dimensionality of the system while preserving information on the variable interactions [26,32]. The basic idea of PCA is to reduce the dimensionality of a dataset in which there are a large number of interrelated variables, while the current variation in the dataset is maintained as much as possible. More specifically, PCA method transforms the original variables into a set of linear combinations, the principal components (PC), which capture the data variability, are linearly independent and weighted in decreasing order of variance coverage. This allows a straightforward reduction of the data dimensionality by discarding the feature elements with low variability. Thus, all original M-dimensional data patterns can be optimally transformed to data patterns in a feature space with lower dimensionality.

The PCA approach is conceptually and computationally quite simple. Given matrix $G=\left(x_{ij}\right)$, where $x_{ij}$ denotes the feature value of sample j for feature i, such that i=1,2,...,M and j=1,2,...,N. Firstly, the M-dimensional means vector $u_{j}$ and M×M covariance matrix Σ are computed for the full dataset. Next, the eigenvectors and eigenvalues are computed, and sorted according to decreasing eigenvalue. Call these eigenvectors $e_{1}$ with eigenvalue $\lambda_{1}$, $e_{2}$ with eigenvalue $\lambda_{2}$, and so on. Next, the largest k eigenvectors are chosen. In practice, this is done by looking at a spectrum of eigenvectors. The largest eigenvalues correspond to the dimensions that explain larger amounts of variance of the dataset. Form a M×k matrix A whose columns consist of the k eigenvectors. Then the k-dimensional feature space (k<M) can be transformed by: $Y=A^{T}G\left(x\right)$. It has been proved that this representation minimizes a squared error criterion.

### Extreme learning machine (ELM)

Feed-forward neural networks (FNN) are ideal classifiers due to their approximation capabilities for nonlinear mappings. However, the slow learning speed of FNN has been a major bottleneck in different applications. The input weights and hidden layer biases of FNN had to be adjusted using some parameter tuning approach such as gradient descent based methods, which are generally time-consuming due to inappropriate learning steps with significantly large latency to converge to a local maxima. In previous works [18,33,34], Huang *et al*. proved that the single hidden layer feed-forward neural networks (SLFNN) could exactly learn N distinct observations for almost any non-linear activation function with almost N hidden nods [18].

Extreme Learning Machine (ELM) was originally developed for the SLFNN and then extended to the generalized SLFNN where the hidden layer need not be neuron alike [18,33]. Its architecture is similar to that of a SLFNN. Recently ELM has been increasingly popular in classification tasks due to its high generalization ability and fast learning speed. Unlike the popular thinking that network parameters need to be tuned, the input weights and first hidden layer biases need not be adjusted but they are randomly assigned in ELM. The ELM algorithm has been proven to perform learning at an extremely fast speed, and obtains good generalization performance for activation functions that are infinitely differentiable in hidden layers.

ELM transforms the learning problem into a simple linear system whose output weights can be analytically determined through a generalized inverse operation of the hidden layer weight matrices. Such a learning scheme can operate at extremely faster speed than learning methods of traditional learning frameworks. Improved generalization performance of ELM with the smallest training error and the norm of weights demonstrate its superior classification capability for real-time applications at an exceptionally fast pace without any learning bottleneck [35].

**Figure 3:**
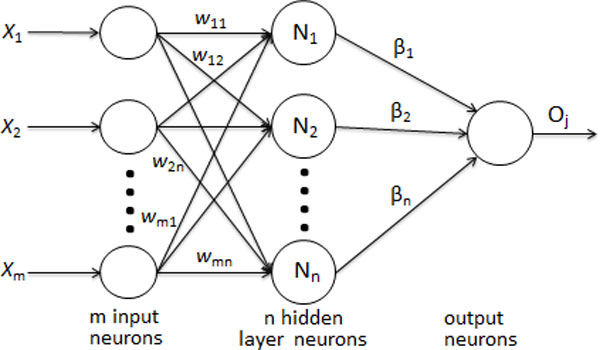
The structure of ELM model

The idea behind ELM is presented as follows: suppose learning N arbitrary different instances $\left(x_{i},t_{i}\right)$, where $x_{i}={\left[x_{i1},x_{i2},...,x_{in}\right]}^{T}\subseteq R^{n}$, $t_{i}={\left[t_{i1},t_{i2},...,t_{im}\right]}^{T}\subseteq R^{m}$, a standard ELM with L hidden neurons and activation function g(x) are mathematically modeled by:

(8) $$\overset{L}{\underset{i=1}{\sum }}\beta_{i}g\left(x_{j}\right)=\overset{L}{\underset{i=1}{\sum }}\beta_{i}g\left(w_{i}\cdot x_{j}+b_{i}\right)=o_{j}{,}_{}j=1,...,N$$

where $w_{i}={\left[w_{i1},w_{i2},...,w_{in}\right]}^{T}$ represents the weight vector connecting the ith hidden node and the input nodes, $\beta_{i}={\left[\beta_{i1},\beta_{i2},...,\beta_{im}\right]}^{T}$ represents the weight vector connecting the ith hidden neuron and the output neurons, and $b_{i}$ denotes the threshold of the ith hidden neuron. $w_{i}\cdot x_{j}$ denotes the inner product of $w_{i}$ and $x_{j}$. The architecture of ELM is shown in Figure 3. The above modeled ELM can reliably approximate these N samples with zero error, which means that $\overset{N}{\underset{j=1}{\sum }}∥o_{j}-t_{j}∥=0$, i.e., there exist $\beta_{i}$, $w_{i}$ and $b_{i}$ such that

(9) $$\overset{L}{\underset{i=1}{\sum }}\beta_{i}g\left(w_{i}\cdot x_{j}+b_{i}\right)=t_{j}{,}_{}j=1,...,N$$

The above N equations can be written compactly as:

(10) Hβ=T

where

(11) $$\begin{array}{c} \text{H}\left(w_{1},...,w_{L},b_{1},...,b_{L},x_{1},...,x_{N}\right)\\ ={\left[\begin{array}{ccc} g\left(w_{1}\cdot x_{1}+b_{1}\right) & \cdots \; & g\left(w_{L}\cdot x_{1}+b_{L}\right)\\ \vdots & \cdots \; & \vdots \\ g\left(w_{1}\cdot x_{N}+b_{1}\right) & \cdots \; & g\left(w_{L}\cdot x_{N}+b_{L}\right) \end{array}\right]}_{N\times L} \end{array}$$

$\beta ={\left[\begin{array}{c} \beta_{1}^{T}\\ \vdots \\ \beta_{L}^{T} \end{array}\right]}_{L\times m}$ and $\text{T}={\left[\begin{array}{c} t_{1}^{T}\\ \vdots \\ t_{N}^{T} \end{array}\right]}_{N\times m}$

H is termed as the hidden layer output matrix of the SLFNN; the ith column of H is the ith hidden neuron's output vector with respect to inputs ${\text{x}}_{\text{1}}{\text{,}}_{}{\text{x}}_{\text{2}}\text{,}\cdots {\text{,}}_{}{\text{x}}_{\text{N}}$. Hence for fixed arbitrary input weights $w_{i}$ and the hidden layer bias $b_{i}$, training a SLFNN equals to find a least-squares solution $\hat{\beta }$ of the linear system Hβ=T. $\hat{\beta }=H^{†}T$ is the best solution, where $H^{†}$ is the Moore-Penrose inverse method for obtaining good generalization performance with extremely fast learning speed.

The procedure of ELM for single-layer feed-forward networks can be summarized as follows: Given a training dataset $ℵ=\left\{\left(x_{i},t_{i}\right)\left|x_{i}\in R^{n},\right)t_{i}\in R^{m},i=1,\cdots \;,N\right\}$, activation function g(x), and hidden neuron number L.

Step 1: Assign arbitrary input weight $w_{i}$ and bias $b_{i}$, i=1,⋯,L.

Step 2: Calculate the hidden layer output matrix H.

Step 3: Calculate the output weight $b_{i}$:

(12) $$\hat{\beta }=H^{†}T$$

where $\hat{\beta }$ and T are defined as formula above.

The learning speed of ELM can be thousands of times faster than traditional feed-forward network learning algorithms like back-propagation (BP) algorithm while obtaining better generalization performance.

The ELM employs a completely different algorithm for calculating weights and biases, unlike the back-propagation or conjugate gradient descent training algorithm. The ELM algorithm is a learning algorithm for single hidden-layer fed-forward networks. The input weights $w_{i}$, and the hidden layer bias are randomly chosen and the output weights $\beta_{i}$ are analytically determined based on the Moore-Penrose generalized inverse of the hidden-layer output matrix. The algorithm is implemented easily and tends to produce a small training error. It also produces the smallest weights norm, performs well and is extremely fast.

### Ensemble of extreme learning machines (E-ELM)

The extreme learning machine training algorithm described above indicates that the randomly initialized hidden layer weights for model accuracy are very important. Therefore, to make results independent of random weights, we train multiple ELMs on the same training dataset, with each having the same number of hidden layer neurons but different randomly assigned weights. Once trained separately, the final output for each sample is determined by combining the outputs of each individual ELM using majority voting strategy. This procedure is usually known as ensembling and the network is called as Ensemble Extreme Learning Machines (EELM). Compared with traditional methods, ensemble classifier can effectively improve classification performance, reliability and stability of individual classifier[36].

## Competing interests

The authors declare that they have no competing interests.

## Authors' contributions

ZY & YL conceived the algorithm, carried out analyses, prepared the data sets, carried out experiments, and wrote the manuscript. LZ & JX & BN designed, performed and analyzed experiments and wrote the manuscript. All authors read and approved the final manuscript.

## Declarations

The publication costs for this article were funded by the corresponding author's institution.

This article has been published as part of *BMC Bioinformatics *Volume 14 Supplement 8, 2013: Proceedings of the 2012 International Conference on Intelligent Computing (ICIC 2012). The full contents of the supplement are available online at http://www.biomedcentral.com/bmcbioinformatics/supplements/14/S8.
